# Effects of cGMP/Akt/GSK-3β signaling pathway on atrial natriuretic peptide secretion in rabbits with rapid atrial pacing

**DOI:** 10.3389/fphys.2022.861981

**Published:** 2022-08-19

**Authors:** Shuxia Cao, Chengyong Han, Chunhua Xuan, Xiangdan Li, Jing Wen, Dongyuan Xu

**Affiliations:** ^1^ Center of Morphological Experiment, Medical College of Yanbian University, Jilin, China; ^2^ Department of Cardiology, Affiliated Hospital of Yanbian University, Jilin, China; ^3^ Key Laboratory of Medical Electrophysiology, Ministry of Education and Medical Electrophysiological Key Laboratory of Sichuan Province, Institute of Cardiovascular Research, Southwest Medical University, Luzhou, China

**Keywords:** rapid atrial pacing, atrial natriuretic peptide, particulate guanylate cyclase, cyclic guanosine monophosphate, natriuretic peptide receptor A, AKT/GSK-3β signaling pathway

## Abstract

Atrial natriuretic peptide (ANP) plays a pivotal role in the regulation of the cardiovascular system. The ANP level increases during atrial fibrillation (AF), suggesting that AF may provoke ANP secretion, but its potential mechanism is still unclear. In the present study, the potential mechanisms of rapid atrial pacing (RAP) regulating ANP secretion was explored. Rabbits were subjected to burst RAP, ANP secretion increased whereas cyclic guanosine monophosphate (cGMP) concentrations decreased during RAP. The p-Akt and p-GSK-3β levels decreased in atrial tissues. Natriuretic peptide receptor A (NPR-A) protein and particulate guanylate cyclase (PGC) activity were detected. The sensitivity of NPR-A to ANP decreased, leading to the decrease of PGC activity. Also, the isolated atrial perfusion system were made in the rabbit model, cGMP was shown to inhibit ANP secretion, and the Akt inhibitor LY294002 (LY) and GSK-3β inhibitor SB216763 (SB) attenuated the inhibitory effects of cGMP on ANP secretion and enhanced the inhibitory effects of cGMP on atrial dynamics. In conclusion, NPR-A interacts with ANP to regulate PGC expression, and influence the expression of cGMP during RAP, which involves in the Akt/GSK-3β signaling pathway. From the aforementioned points we conclude that cGMP regulates ANP secretion by the Akt/GSK-3β signaling pathway during atrial pacing.

## 1 Introduction

Atrial fibrillation (AF) is the most common persistent arrhythmia. From a pathophysiological point of view, the development of AF is accompanied by electrical remodeling, structural remodeling, and autonomic nerve remodeling, and these changes are collectively called “atrial remodeling” (Schotten et al., 2011).

The heart as an endocrine organ can secrete atrial natriuretic peptide (ANP) (Xu et al., 2008), which participates in water and sodium metabolism, maintains blood volume, and regulates arterial blood pressure (Pagel-Langenickel, 2018). ANP secretion is increased during AF. BNP is mainly secreted by the heart as a member of the natriuretic peptide family, a peptide consisting of 32 amino acid residues. It can regulate the stability of blood pressure and blood volume in the body and has a diuretic effect. Both ANP and BNP have an effect on the heart to inhibit cardiac hypertrophy and myocardial fibrosis (Nakagawa et al., 2019). Mechanical stretch stimulation of atrial myocytes is the main factor causing ANP secretion (Bie, 2018), and some hormones, such as angiotensin II (Soualmia et al., 1997), endothelin-1 (Stasch et al., 1989), and adrenergic agonists (Thibault et al., 1999), can stimulate ANP secretion.

The Akt/GSK-3β signaling pathway is known to be critically involved in various pathological processes of the heart (Liu et al., 2020), including myocardial fibrosis (Qin et al., 2021), myocardial hypertrophy (Dorn and Force, 2005), and ischemia-reperfusion injury (Mocanu and Yellon, 2007). Akt affects the development of heart failure by participating in energy metabolism (Sugiyama et al., 2019), resulting in myocardial remodeling and apoptosis. In addition, GSK-3β as a downstream molecule of Akt, participates in other physiological activities in the human body, including DNA repair and apoptosis (Lin et al., 2020).

Cyclic guanosine monophosphate (cGMP), as a second messenger, negatively regulates the secretion of ANP (Lee et al., 2000), and the cGMP-mediated Akt/GSK-3β signaling pathway has been shown to be closely related to cardiac electrophysiology, myocardial hypertrophy, and myocardial contraction (Bork et al., 2019). However, the mechanisms between cGMP, Akt/GSK-3β pathway, and ANP during AF are not clear.

In this study, we explored the effects of cGMP-mediated ANP secretion by the Akt/GSK-3β pathway in a rabbit RAP model to elucidate the pathophysiological mechanisms of cardiac endocrine changes in AF.

## 2 Materials and methods

### 2.1 Animal preparation

The experimental animals were male New Zealand white rabbits (Department of Zoology, Yanbian University; weight: 2.5–3 kg; *n* = 6 rabbits per group). The rabbits were anesthetized with 20% urethane (5 ml/kg) *via* the ear vein. The right external jugular vein was identified along the lower edge of the sternocleidomastoid muscle, and a “V” incision was made in the right external jugular vein. An electrode catheter was inserted into the right atrium along the right external jugular vein, and a J-shaped guidewire was inserted into the sleeve. The guidewire was slowly pushed out of the sleeve and fixed. The J-type guidewire is connected to the cathode of the electrode to stimulate the atrium. The electrode anode is implanted at the strongest beating of apex cordis as a reference electrode. Aatrial beating is stimulated by monopolar pacing. Animal experiments did comply with the ARRIVE guidelines, and all experimental protocols were approved by the animal ethics committee of Yanbian University. The secretion of ANP involves endocrine processes, and its secretion is related to the regulation of endocrine secretion. In contrast, most female mammals have a physiological cycle, in which there is a relationship between the physiological cycle and ANP secretion. It is difficult for us to control their physiological cycle, in order to avoid the disturbance of ANP secretion by the presence of the physiological cycle, we have studied the mechanism of ANP secretion using male animals. So the results from this study are focused on male rabbits, and female rabbits remain to be further investigated.

### 2.2 Preparation of the rabbit rapid atrial pacing model

**FIGURE 1:**
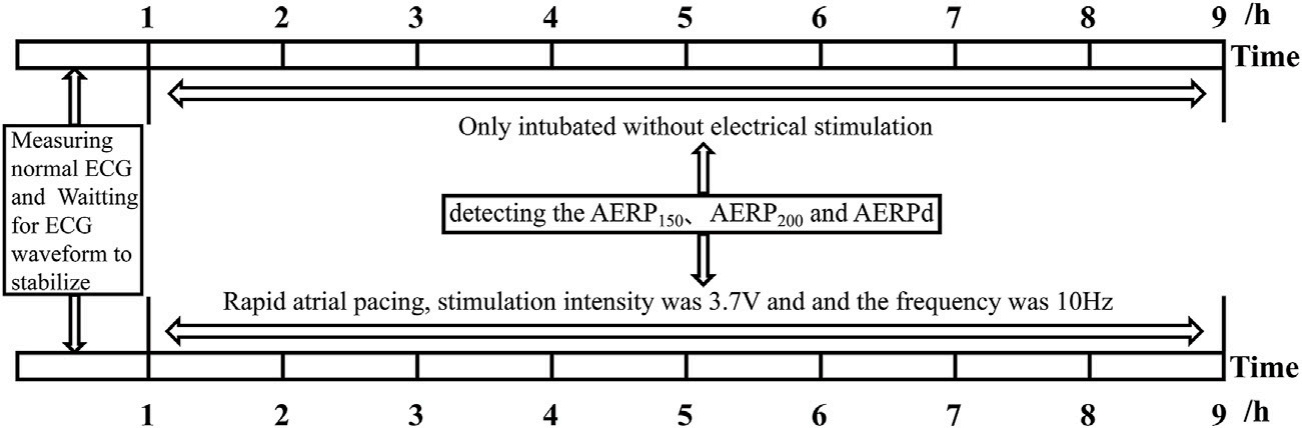
Experimental protocols *in vivo* of rabbit.

After preparation of the aforementioned experimental animals, an RM6240 physiological function experimental system (RM6240BD; Chengdu, Sichuan, China) was used to connect the limb leads and detect the normal ECG waveform. After stabilization, the positive electrode was connected to the J-shaped guidewire and subjected to electrical stimulation. The stimulation intensity was 3.7 V, the pulse width was 0.5 ms, the delay was 0 s, and the frequency was 10 Hz. The animals were randomly divided into two groups: the control group (P0, only intubated without electrical stimulation, *n* = 6) and the rapid pacing 8 h group (P8, *n* = 6). The atrial effective refractory period (AERP) was measured using an electrophysiological instrument with the S_1_S_2_ program incremental stimulation method. The S_1_S_2_ refractory period was scanned incrementally (8:1, step size: 2 ms), and the longest S_1_S_2_ interval that could not be transmitted down to the atrium was considered the AERP. AERP_150_ and AERP_200_ in the control and pacing groups were detected (the circumferences of S_1_S_2_ basic stimulation were 150 and 200 ms, respectively) (Figure 1).

### 2.3 Preparation of the isolated beating perfused rabbit atrial model

For preparation of the isolated beating perfused rabbit atrial model, New Zealand white rabbits were used (*n* = 6 rabbits/group). Briefly, 20% urethane (5 ml/kg) was injected intravenously through the ear margin for anesthesia, and the isolated beating perfused rabbit atrial model was prepared as described previously (Cho et al., 1993). For this model, the abdominal cavity was opened, and the heart was removed and rinsed in normal saline. The left atrium was removed and quickly fixed using an atrial perfusion device developed by Cho et al. (1995). Next, the atrium inserted into the cannula was placed into a perfusion cylinder containing HEPES buffer (36.5°C). Oxygen was delivered to the irrigation fluid through a silicone tube on the device. HEPES buffer was perfused into the atrium at a flow rate of 1 ml/min using a peristaltic pump. The composition of the buffer was as follows: distilled water, 118 mM·NaCl, 4.7 mM KCl, 2.5 mM CaCl_2_, 1.2 mM MgCl_2_, 25 mM NaHCO_3_, 10 mM glucose, and 0.10% bovine serum albumin. Electrode stimulation was applied through the atrial wall immediately after perfusion, with the following stimulation parameters: voltage, 35 V; wave width, 0.5 ms; low-frequency, 1.5 Hz; and high frequency, 4.0 Hz. The atrial pulse pressure was measured using a pressure sensor connected to the atrial catheter and recorded in an RM6240 biological signal acquisition system. The atrial stroke volume was calculated based on the change in the water column of the transparent cannula during atrial contraction.

### 2.4 Experimental protocols

Before the formal experiment, each atrium was perfused for 60 min to stabilize ANP secretion. The perfusate was collected at 2 min intervals at 4°C. The experiment cycle was 12 min.

**FIGURE 2:**
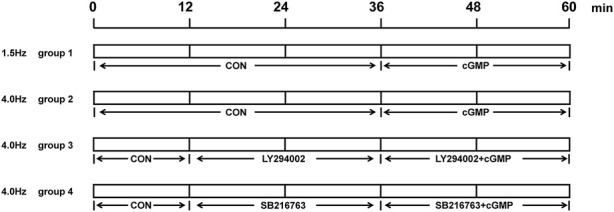
Experimental protocols *in vitro*. The order and time of adding of perfusion reagents for each group in the isolated perfusion experiment.

Animals were divided into four groups (*n* = 6 rabbits/group; Figure 2), as follows: first, the low-frequency stimulation group was subjected to 1.5 Hz stimulation. The perfusate was collected as a control for the first 36 min. 8-Br-cGMP (1 mmol/L, ab141449, abcam) was then added, and the perfusate was collected for an additional 24 min. Then, the high-frequency stimulation group was subjected to 4.0 Hz stimulation. The perfusate was collected as a control for the first 36 min. 8-Br-cGMP was added, and the perfusate was collected for an additional 24 min. Next, for the high-frequency stimulation plus Akt inhibitor LY294002 group, the perfusate was collected as a control during the first 12 min. LY294002 (10 μmol/L, ab120243, Abcam) was added, and the perfusate was collected for 24 min. 8-Br-cGMP was added, and the perfusate was collected for 24 min. Finally, for the high-frequency stimulation plus GSK-3β inhibitor SB216763 group, the perfusate was collected as a control during the first 12 min. SB216763 (10 μmol/L, ab120202, Abcam) was added, and the perfusate was collected for 24 min. 8-Br-cGMP was added, and the perfusate was collected for 24 min.

### 2.5 Hematoxylin and eosin staining

After the RAP model was made, the atrial tissue from rabbits was removed immediately, fixed in 10% formalin fixation solution for 24 h, and then rinsed with tap water for 12 h. Alcohol gradient dehydration was performed, and the heart tissues were then embedded in paraffin, cut into 4-μm thick sections, and baked in an oven at 56°C overnight. The sections were then stained using an H&E staining kit (Beijing Solarbio Technology Co., Ltd., Beijing, China).

### 2.6 Transmission electron microscopy

The rabbit’s atrial tissues were removed immediately after the RAP model was made, immersed in a precooled 2.5% glutaraldehyde solution, and cut into 1-mm^3^ tissue blocks, fixed in 2.5% glutaraldehyde for 2 h. Subsequently, samples were washed with precooled phosphate-buffered saline (PBS) three times, fixed with osmic acid for 2 h, and washed again with precooled PBS three times. The samples were then dehydrated with 50%, 70%, and 90% ethanol at 4°C for 30 min and penetrated with 90 and 100% acetone. Finally, the samples were embedded, sliced at a thickness of 70 nm, and stained with 3% uranium acetate lead citrate. Transmission electron microscopy was performed.

### 2.7 Enzyme-linked immunosorbent assay of cGMP

After 8 h of electrical stimulation, the atrial tissue from rabbits was removed immediately, and the atrial tissue was cut up. The atrial tissue was homogenized at 4°C by three 30-s bursts of maximal speed using a tissue tearor, and then centrifuged at 16,000 rpm at 4°C for 10 min. The supernatant was collected, and then ELISA was performed using an ELISA kit (Nanjing Sbjbio Biotechnology Co., Ltd., Nanjing, China), according to the manufacturer’s instructions. The absorbance was measured at 450 nm by an enzyme-labeling instrument. The cGMP concentration in atrial tissue was calculated according to the absorbance of cGMP based on a standard curve.

### 2.8 Radioimmunoassay of atrial natriuretic peptide

ANP standard, ^125^I-ANP, and rabbit anti-ANP antibodies were dissolved and diluted with buffer from a ^125^I-ANP radioimmunoassay kit (Beijing North Institute of Biology Co., Ltd., Beijing, China). ANP standard substance was dissolved with ANP buffer, according to ^125^I-ANP radioimmunoassay kit instructions and a solution with a final concentration of 4.0 ng/ml was obtained, and dilution was made (4.0, 2.0, 1.0, 0.5, and 0.125 ng/ml). 1) Number the special test tubes, total T (total tracer test tubes), NBS (non-specific immune test tubes), S0 (control tubes), S1-6 (standard test tubes), and sample tubes to be tested. 2) Before adding sample, all samples to be tested and ^125^I-ANP, rabbit anti-ANP antibody, buffer solution, and other reagents were mixed at 4°C, respectively. Added samples are as shown in the table (Table 1). 3) After the samples were added, they were mixed and incubated in a refrigerator at 4°C for 16–24 h. 4) All tubes were added with 500 μl of donkey antirabbit immune isolate except the total T. After incubating for 15 min at room temperature, centrifuge at 4°C for 18 min at 3,500 rpm and discard the supernatant. The radioactivity in each sediment was detected by using a counter.

**TABLE 1:** RIA of ANP.

Tube type	Total T	NBS	S0 tube	S1–6 tube	Sample tube
Reagent					
ANP buffer	0	200 μl	100 μl	0	75 μl
ANP standard substance	0	0	0	100 μl	0
Sample to be tested	0	0	0	0	25 μl
^125^I-ANP	100 μl	100 μl	100 μl	100 μl	100 μl
Rabbit anti-ANP antibody	0	0	100 μl	100 μl	100 μl

### 2.9 Measurement of particulate guanylate cyclase activity

The atrial tissues of rabbits in the control group and the rapid pacing stimulation group were collected right after the RAP model was made. The atrial tissue was placed in ice-cold phosphate buffer, and the atrial tissue was cut up. The atrial tissue was homogenized at 4°C by three 30 s bursts of maximal speed using a tissue tearor. The homogenate was centrifuged at 1,000 g for 10 min at 4°C, and supernatant collected. The supernatant was recentrifuged at 40,000 g for 60 min at 4°C, and precipitation was collected (membrane pellet). The membrane pellet was washed three times with Tris-HCl buffer containing EDTA. Protein contents were determined by a BCA kit (Thermo Fisher Scientific, MA, United States). After the assay was completed, it was divided into equal amounts of protein suspensions. Then aliquots of the protein suspension were incubated for 15 min at 37°C in Tris-HCl buffer, containing 3-isobutyl-1-methylxanthine (IBMX, Sigam I5879), GTP, ATP, creatine phosphate, creatine phosphokinase, MgCl_2_, and ANP. Incubations were stopped by adding ice-cold sodium acetate and boiling for 5 min. The samples were then centrifuged at 10,000 g for 5 min at 4°C. ANP was added to the experimental group, and only Tris-HCl buffer was added to the blank group as the control group.

### 2.10 Western blotting

A total protein extraction kit (Millipore, Billerica, MA, United States) was used to obtain total protein from atrial tissues. BCA working solution (Thermo Fisher Scientific, MA, United States) was used to determine protein concentrations. Bromophenol blue was added, and the samples were boiled to denature the protein. Proteins were then separated by SDS-PAGE gel electrophoresis and transferred to polyvinylidene fluoride membranes. Membranes were blocked with 5% skim milk powder for 1.5 h and then incubated overnight at 4°C with the following primary antibodies: anti-NPR-A [1:1000], anti-VASP [1:1000], anti-p-VASP [(Ser 239) 1:1000], anti-Akt [1:1000], anti-p-Akt [(Thr 308) 1:1000], anti-GSK-3β [1:1000], anti-p-GSK-3β [ (Ser 9) 1:1000], and anti-β-actin [1:5000]; all from Santa Cruz Biotechnology, Santa Cruz (CA, United States). The membranes were then washed with TBST and incubated with HRP-secondary antibodies at room temperature for 1.5 h. Finally, images were acquired using enhanced chemiluminescence solution.

### 2.11 Statistical analysis

ImageJ software was applied for analysis of images. Data were statistically analyzed using GraphPad Prism software. Statistical significance was evaluated by *t*-tests, one-way ANOVA, and tow-way ANOVA. Data were presented as mean ± SE. Results with *p* < 0.05 were considered significant.

## 3 Results

### 3.1 Induction of rapid atrial pacing

**FIGURE 3:**
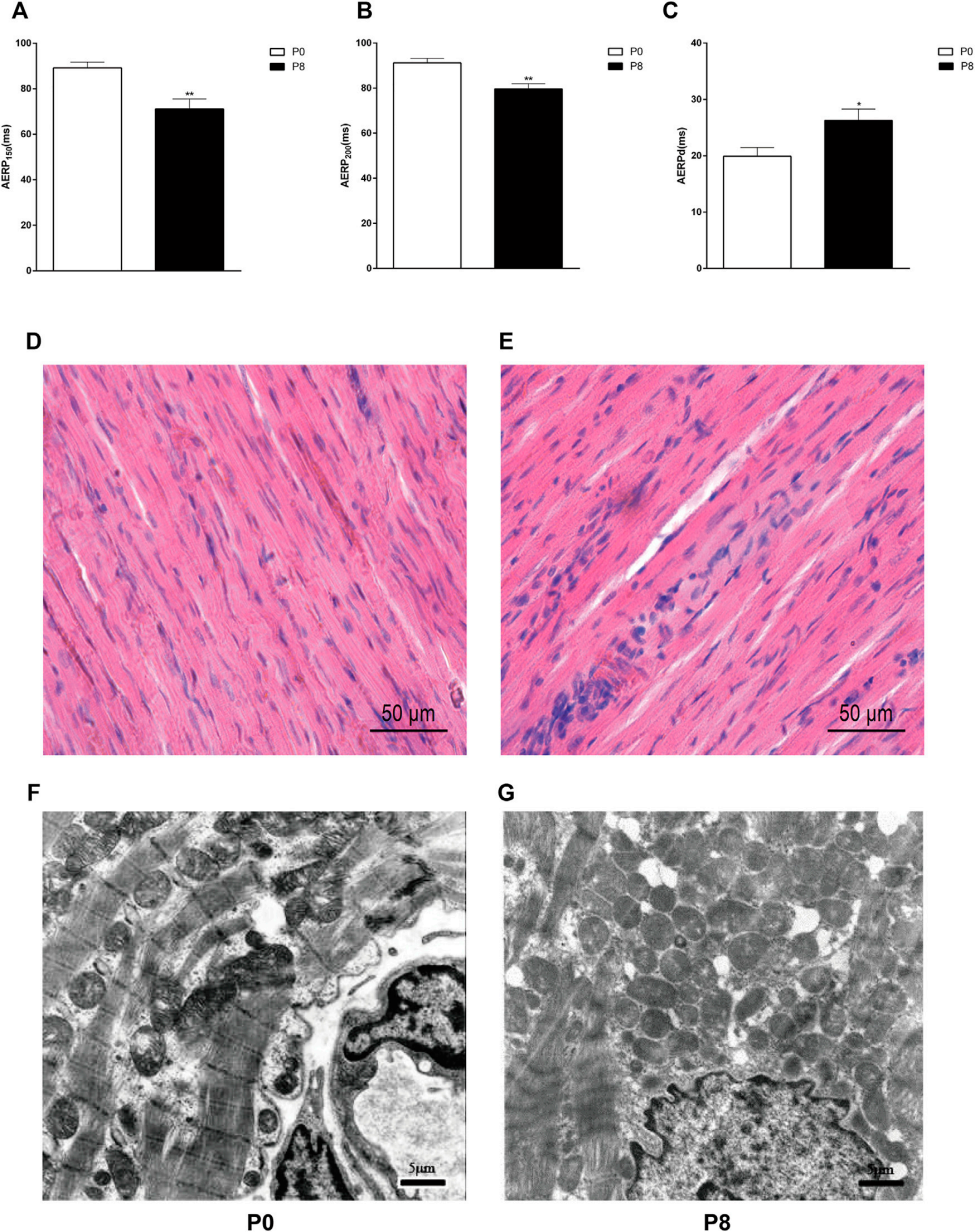
Rapid pacing rabbit atrial model was successfully established. Changes in AERP_150_ **(A)**, AERP_200_ **(B)**, and AERPd **(C)** after rapid pacing stimulation. **p < 0.01 vs. P0; *p < 0.05 vs. P0. Effects of rapid pacing on the morphological structure and atrial ultrastructure of atrial muscles in rabbits. **(D)** Cell structure was complete, and the myocardial fiber was evenly distributed and arranged vertically and horizontally. The atrial tissue is from the right atrial of P0. **(E)** Cell boundary distribution was unclear. The arrangements of myocardial fiber were disordered, and the normal network structure was lost and leukocyte infiltration was also observed. The atrial tissue is from the right atrial of P8. **(F)** Ultrastructure of cardiomyocytes in P0 is intact. **(G)** P8 showed incomplete nuclear membranes, disappearance of gap links, expansion of the sarcoplasmic reticulum, and discontinuity of the bilayer membrane. Data are expressed as mean ± SEMs. *n* = 6.

The forelimbs of rabbits trembled with electrode stimulation, and incontinence occurred during the late stages of the experiment. AERP_150_ and AERP_200_ were significantly shorter in the pacing group than that in the control group (Figures 3A,B), whereas AERPd increased (Figure 3C). Meanwhile, the changes in the heart structure in rabbits (Figures 3D–G) were confirmed. The cell structure was complete, and the myocardial fiber was evenly distributed and arranged vertically and horizontally (Figure 3D). The cell boundary distribution was unclear. The arrangements of myocardial fiber were disordered, and the normal network structure was lost and leukocyte infiltration was also observed (Figure 3E). The ultrastructure of cardiomyocytes in the P0 is intact (Figure 3F). The P8 showed incomplete nuclear membranes, disappearance of gap links, expansion of the sarcoplasmic reticulum, and discontinuity of the bilayer membrane (Figure 3G).

### 3.2 Rapid atrial pacing influences atrial natriuretic peptide secretion and cGMP concentration in rabbits

**FIGURE 4:**
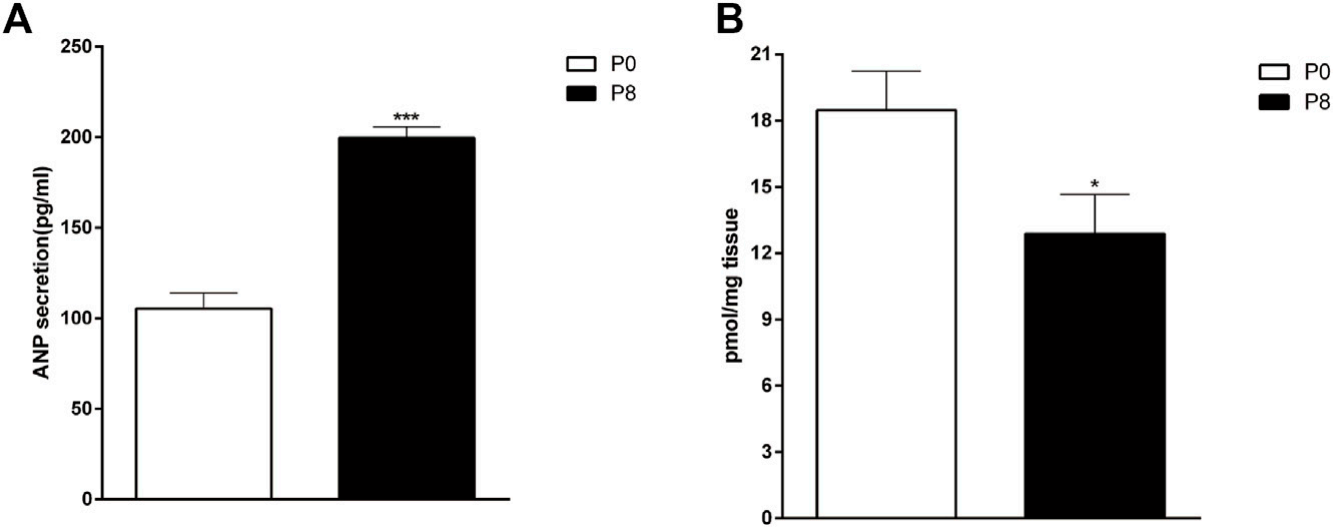
Effects of rapid pacing on atrial ANP secretion and cGMP concentrations in rabbits. **(A)** Changes in ANP secretion **(A)** and cGMP concentrations **(B)** after 8 h of rapid pacing stimulation. ***p < 0.001 vs. P0. Data are expressed as means ± SEMs. *n* = 6.

To determine the influences of RAP on ANP and cGMP in the rabbit atrium, we detected changes in ANP and cGMP by RIA and ELISA. Compared with the P0, secretion of atrial ANP increased significantly in the P8 (Figure 4A), whereas the concentration of cGMP decreased significantly (Figure 4B). Thus, rapid pacing stimulated ANP secretion and inhibited cGMP secretion in rabbits.

### 3.3 NPR-A and particulate guanylate cyclase activity change during rapid atrial pacing

**FIGURE 5:**
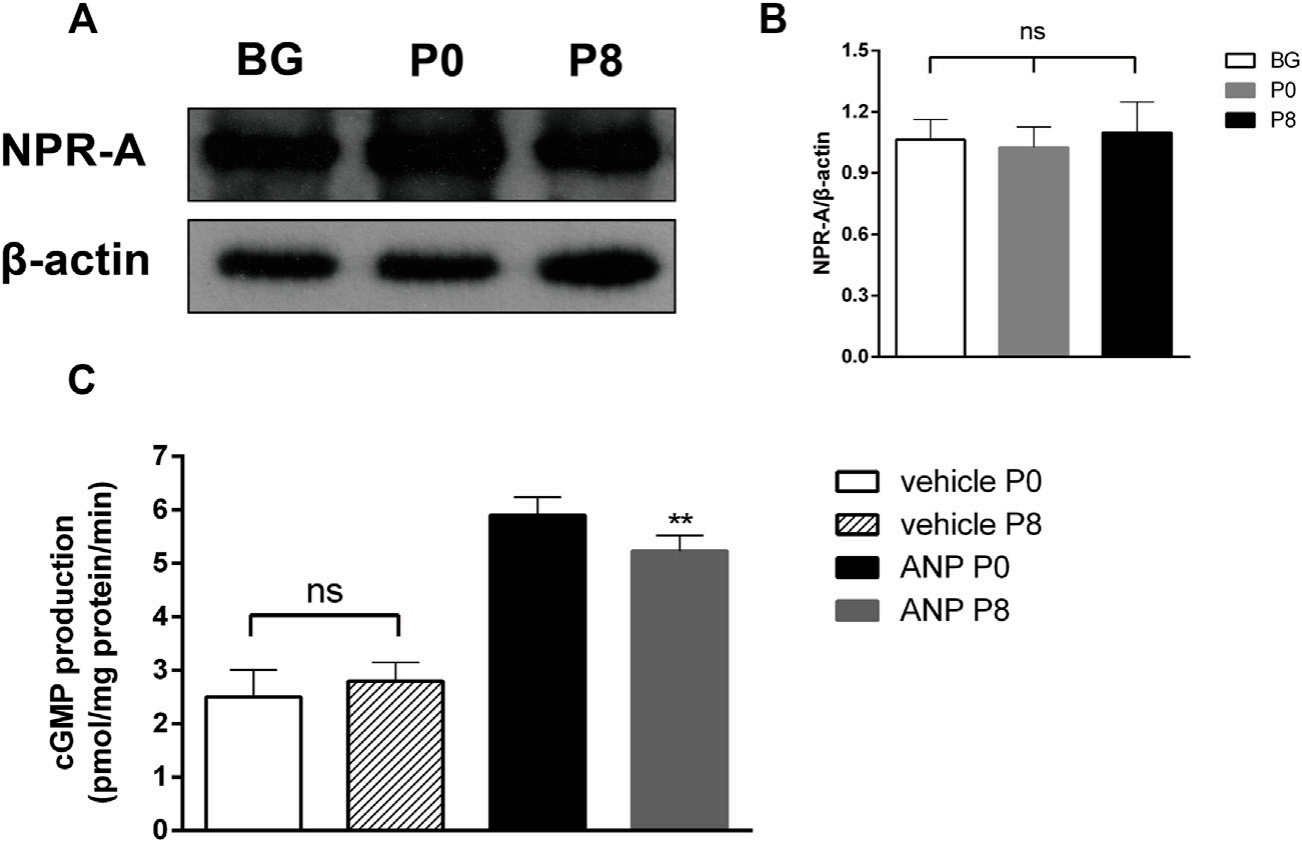
Effects of rapid pacing on NPR-A and PGC activity in the rabbit atrium. **(A)** NPR-A protein level after rapid pacing stimulation for 8 h (BG = Black group). **(B)** Quantitative analysis of NPR-A protein levels, *p* > 0.05. **(C)** cGMP production in the atrial membrane (PGC activity), **p < 0.01 vs. ANP P0. Data are expressed as mean ± SEMs, *n* = 6.

Studies have shown that ANP combined with NPR-A to activate PGC to increase the expression of cGMP (Moghtadaei et al., 2016). To test the NPR-A level in the rabbit atrium during RAP, we detected the NPR-A protein level using Western blotting. The results showed that there was no difference in the level of NPR-A between P0 and P8 (Figures 5A,B). We further investigated the cGMP production in the atrial membrane by RIA and found that the cGMP production among P8 groups decreased in the ANP group, though there was no significant difference between P0 and P8 in the vehicle group (Figure 5C). The production of cGMP relates to the activity of PGC. It showed that the PGC activity is decreased when the sensitivity of NPR-A to ANP is reduced.

### 3.4 Influences of rapid atrial pacing on the Akt/GSK-3β signaling pathway in the rabbit atrium

**FIGURE 6:**
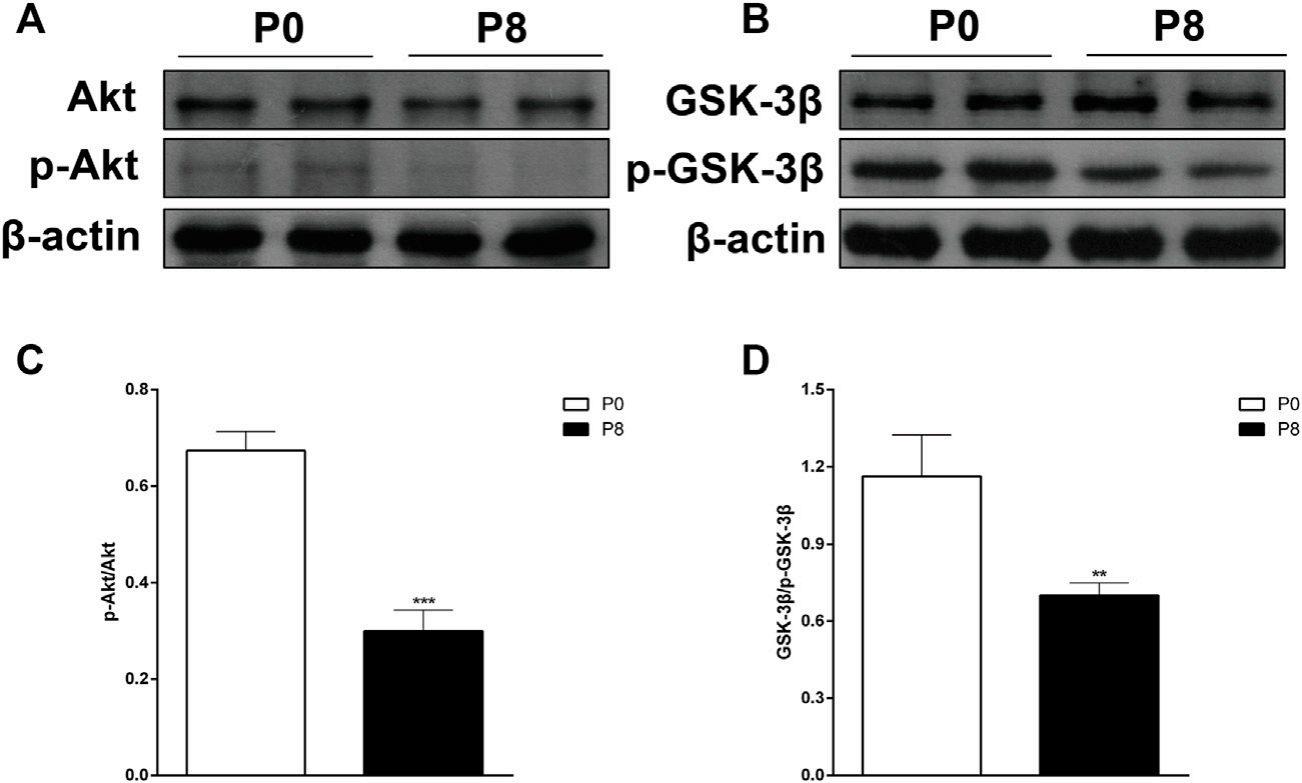
Effects of rapid pacing on Akt/GSK-3β in the rabbit atrium. **(A)** Akt and p-Akt protein levels after rapid pacing stimulation for 8 h. **(B)** GSK-3β and p-GSK-3β protein levels after rapid pacing stimulation for 8 h. **(C)** Quantitative analysis of p-Akt protein levels, ***p < 0.001 vs. P0. **(D)** Quantitative analysis of p-GSK-3β protein level, **p < 0.01 vs. P0. Data are expressed as mean ± SEMs, *n* = 6.

To determine the influences of rapid pacing on the Akt/GSK-3β signaling pathway, we detected the Akt/p-Akt and GSK-3β/p-GSK-3β protein levels using Western blotting. We found that the phosphorylation levels of Akt and GSK-3β decreased significantly (Figure 6) among the atrium of rabbits in P8 compared with that in P0.

### 3.5 High-frequency stimulation affects atrial natriuretic peptide secretion and atrial dynamics in isolated atrial perfusion

**FIGURE 7:**
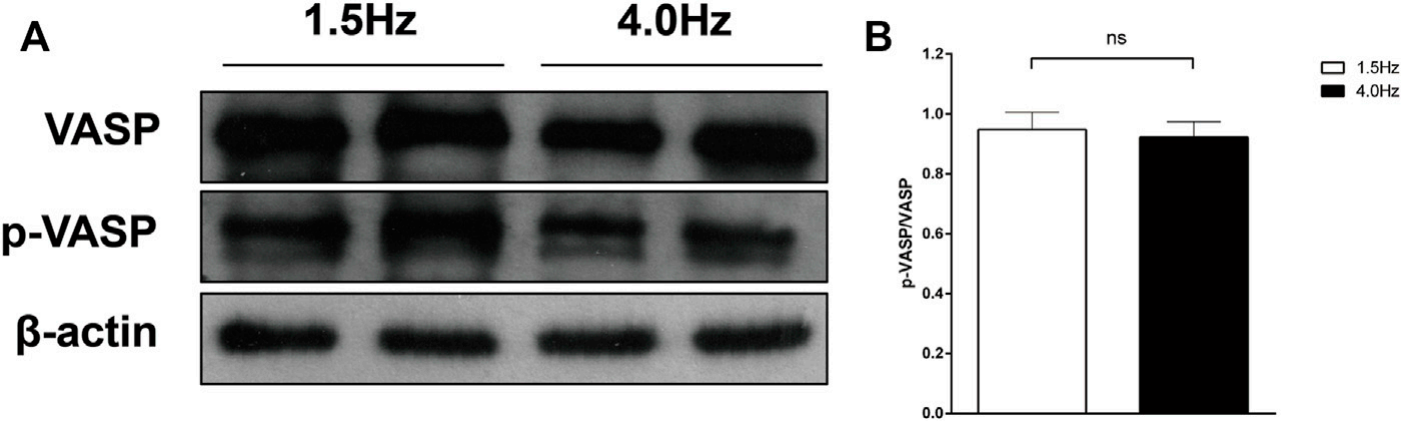
VASP-phosphorylation as indicator of cGMP activity. **(A)** Effect of different stimulation on VASP and p-VASP. **(B)** Quantitative analysis of p-VASP protein levels, *p* > 0.05. Data are expressed as mean ± SEMs, *n* = 6.

**FIGURE 8:**
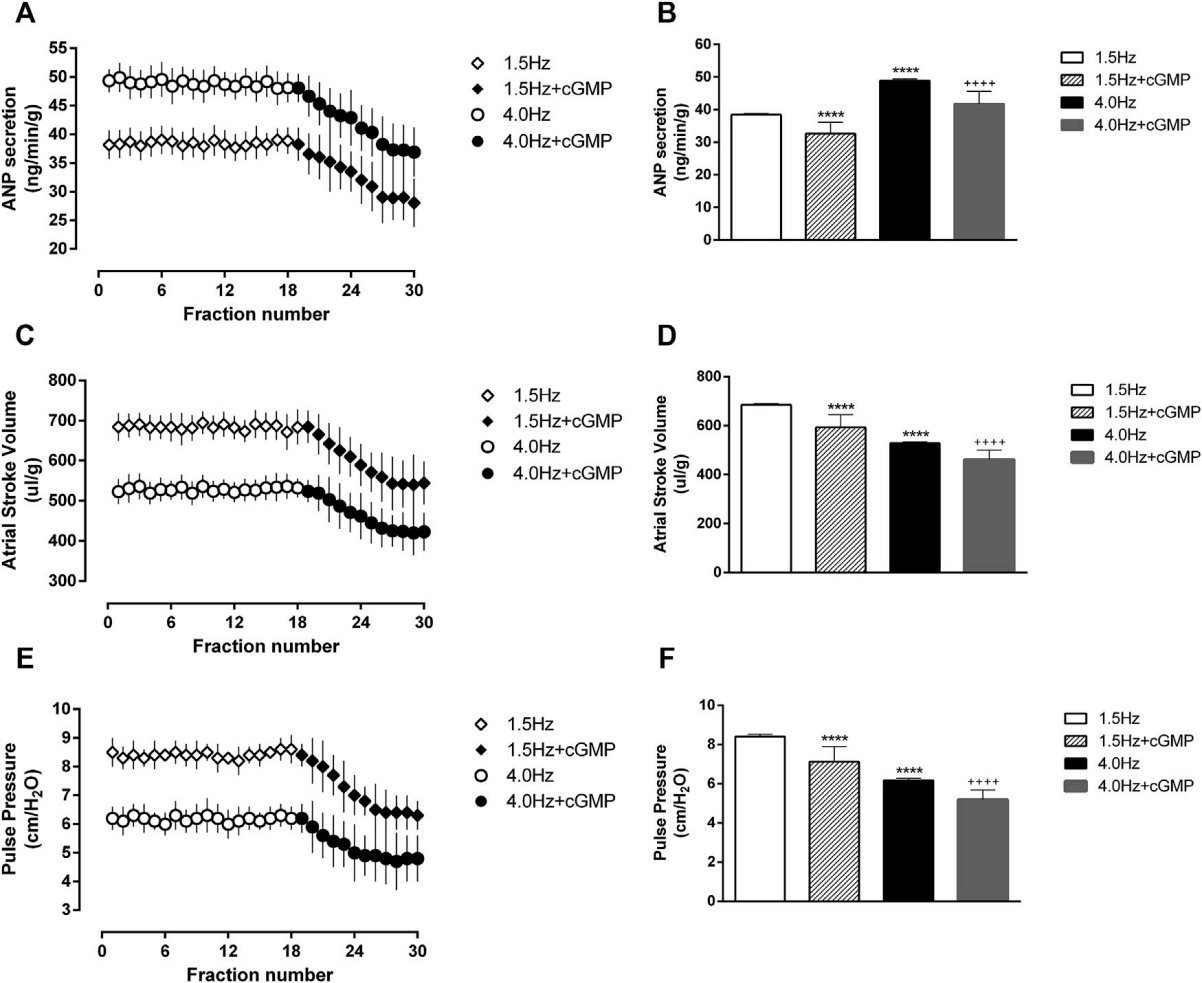
Effects of high-frequency stimulation on ANP secretion and atrial dynamics in isolated atrial perfusion. **(A)** Effects of high-frequency stimulation and cGMP on atrial ANP secretion. **(B)** Quantitative analysis of the effects of high-frequency stimulation and cGMP on atrial ANP secretion. ****p < 0.0001 vs. 1.5 Hz; ^++++^ *p* < 0.0001 vs. 4.0 Hz. **(C)** Effects of high-frequency stimulation and cGMP on atrial stroke volume. **(D)** Quantitative analysis of the effects of high-frequency stimulation and cGMP on atrial stroke volume, ****p < 0.0001 vs. 1.5 Hz; ^++++^ *p* < 0.0001 vs. 4.0 Hz. **(E)** Effects of high-frequency stimulation and cGMP on atrial pulse pressure. **(F)** Quantitative analysis of the effects of high-frequency stimulation and cGMP on atrial pulse pressure, ****p < 0.0001 vs. 1.5 Hz; ^++++^ *p* < 0.0001 vs. 4.0 Hz. Data are expressed as means ± SEMs, *n* = 6.

Frist, we measured VASP-phosphorylation as indicator of cGMP activity (Figure 7). In the isolated atrial perfusion model, the secretion of ANP in the perfusion fluid was detected by radioimmunoassay. Shown in Figures 8A,B are significant increase of ANP with high-frequency stimulation, while ANP secretion was inhibited after adding cGMP. Also shown in Figures 8C,D are the atrial stroke volume high-frequency stimulation reduced with high-frequency stimulation, and adding cGMP could further decrease the atrial stroke volume. In addition, the atrial pulse pressure was reduced by high-frequency stimulation and was further reduced by adding cGMP (Figures 8E,F).

### 3.6 Effects of LY294002 on cGMP-induced atrial natriuretic peptide secretion and atrial dynamics under high-frequency stimulation

**FIGURE 9:**
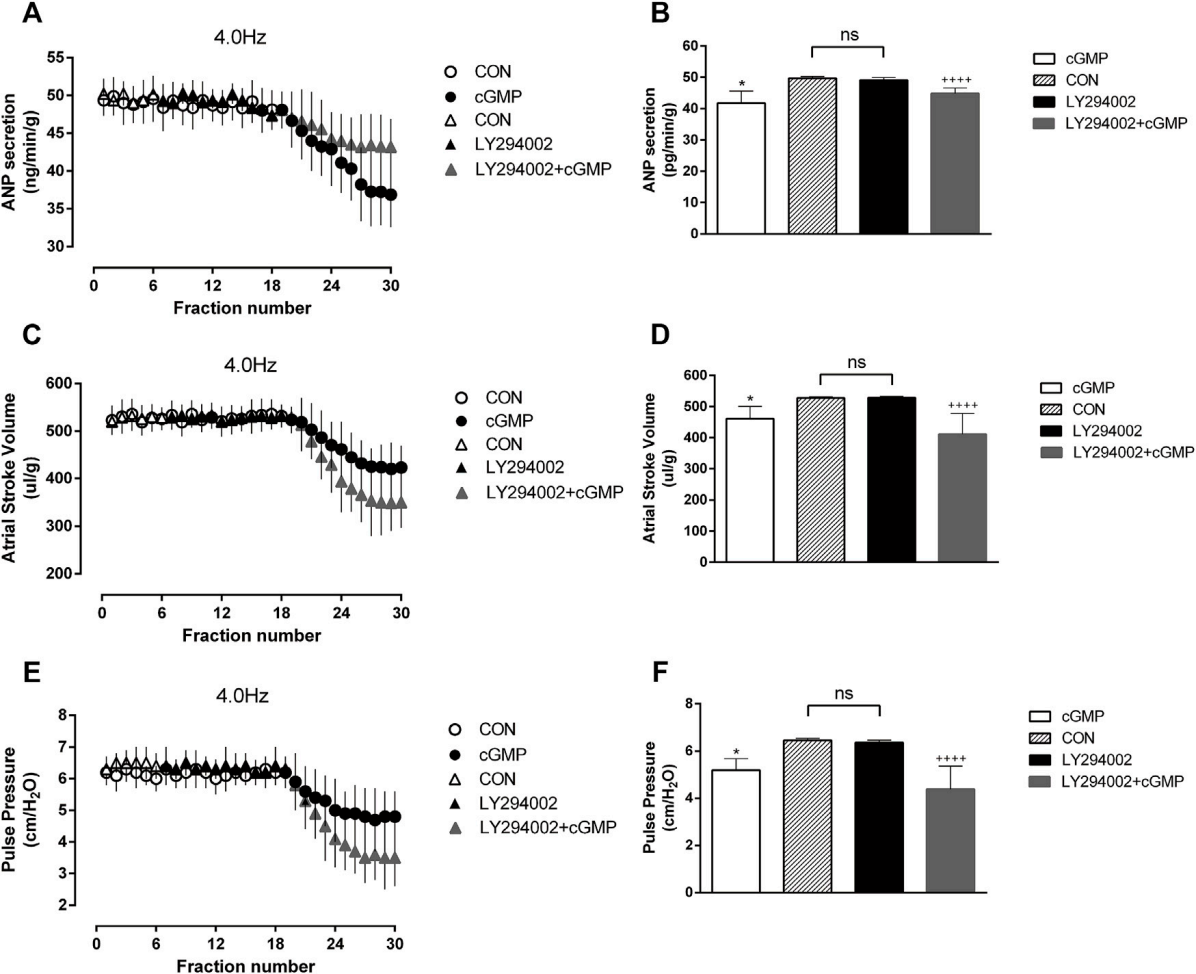
Effects of LY294002 on cGMP-induced ANP secretion and atrial dynamics under high-frequency stimulation. **(A)** Effects of the Akt inhibitor LY on ANP secretion under high-frequency stimulation. **(B)** Quantitative analysis of the effects of LY on ANP secretion under high-frequency stimulation. ^*^ *p* < 0.05 vs. LY + cGMP; ^++++^ *p* < 0.0001 vs. LY. **(C)** Effects of LY on atrial stroke volume under high-frequency stimulation. **(D)** Quantitative analysis of the effects of LY on atrial stroke volume under high-frequency stimulation. *p < 0.05 vs. LY + cGMP; ^++++^ *p* < 0.0001 vs. LY. **(E)** Effects of LY on atrial pulse pressure under high-frequency stimulation. **(F)** Quantitative analysis of the effects of LY on atrial pulse pressure under high-frequency stimulation. *p < 0.05 vs. LY + cGMP; ^++++^ *p* < 0.0001 vs. LY. Data are expressed as mean ± SEMs, *n* = 6. (PS CON and cGMP are from Figure 7).

To examine whether the Akt inhibitor LY294002 affects cGMP function, we made the isolated atrial perfusion model, LY294002 was added for two cycles after one cycle of 4.0 Hz stimulation and the perfusion fluid was collected for detection. We found that there were no significant changes in ANP secretion or atrial dynamics after adding LY294002 (Figure 9). Then the Akt inhibitor and cGMP were added for two cycles, and the perfusion fluid was collected for detection. The results showed that LY294002 reduced the inhibitory effects of cGMP on ANP secretion and enhanced the inhibitory effects of cGMP on atrial dynamics (Figure 9).

### 3.7 Effects of SB216763 on cGMP-induced atrial natriuretic peptide secretion and atrial dynamics under high-frequency stimulation

**FIGURE 10:**
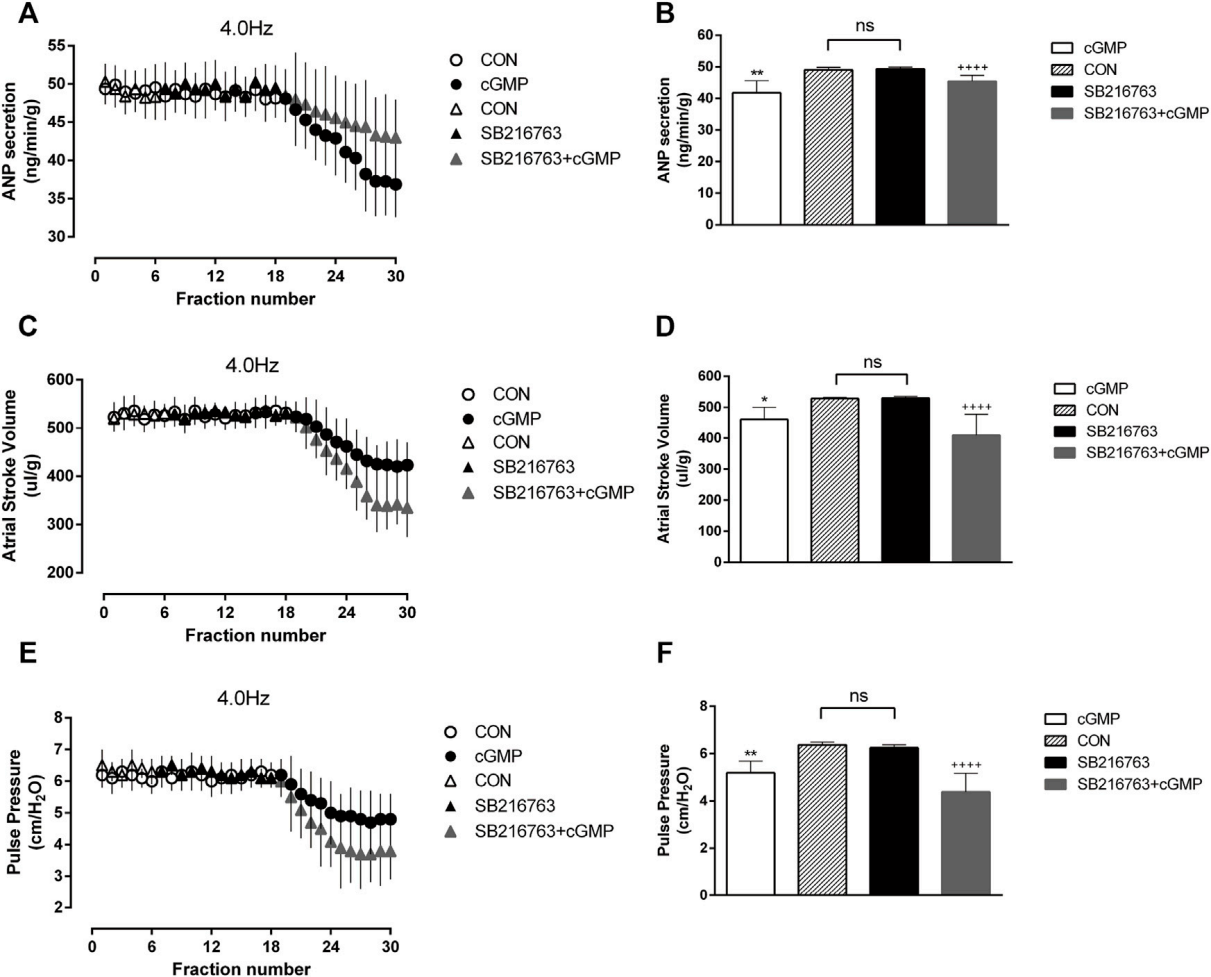
Effects of SB216763 on cGMP-induced ANP secretion and atrial dynamics under high-frequency stimulation. **(A)** Effects of the GSK-3β inhibitor SB on ANP secretion under high-frequency stimulation. **(B)** Quantitative analysis of the effects of SB on ANP secretion under high-frequency stimulation. **p < 0.01 vs. SB + cGMP; ^++++^ *p* < 0.0001 vs. SB. **(C)** Effects of GSK-3β inhibitor SB on atrial stroke volume under high-frequency stimulation. **(D)** Quantitative analysis of the effects of SB on atrial stroke volume under high-frequency stimulation. *p < 0.05 vs. SB + cGMP; ^++++^ *p* < 0.0001 vs. SB. **(E)** Effects of SB on atrial pulse pressure under high-frequency stimulation. **(F)** Quantitative analysis of the effects of SB216763 on atrial pulse pressure under high-frequency stimulation. **p < 0.01 vs. SB + cGMP; ^++++^ *p* < 0.0001 vs. SB. Data are expressed as mean ± SEMs, *n* = 6. (PS CON and cGMP are from Figure 7).

We also performed another test to determine the effects of the GSK-3β inhibitor SB216763 as GSK-3β is the downstream of Akt. The isolated atrial perfusion model was made, and the GSK-3β inhibitor SB216763 was added for two cycles after one cycle of 4.0 Hz stimulation, then the perfusion fluid was collected for detection. No significant changes in ANP secretion or atrial dynamics after the addition of SB are shown in Figure 10 . We next add SB216763 and cGMP for two cycles, then collected the perfusion fluid for detection. We found that SB216763 blocked the inhibitory effects of cGMP on ANP secretion and enhanced the inhibitory effects of cGMP on atrial dynamics (Figure 10).

### 3.8 High-frequency stimulation influences cGMP/Akt/GSK-3β signaling pathway in isolated perfused atrium

**FIGURE 11:**
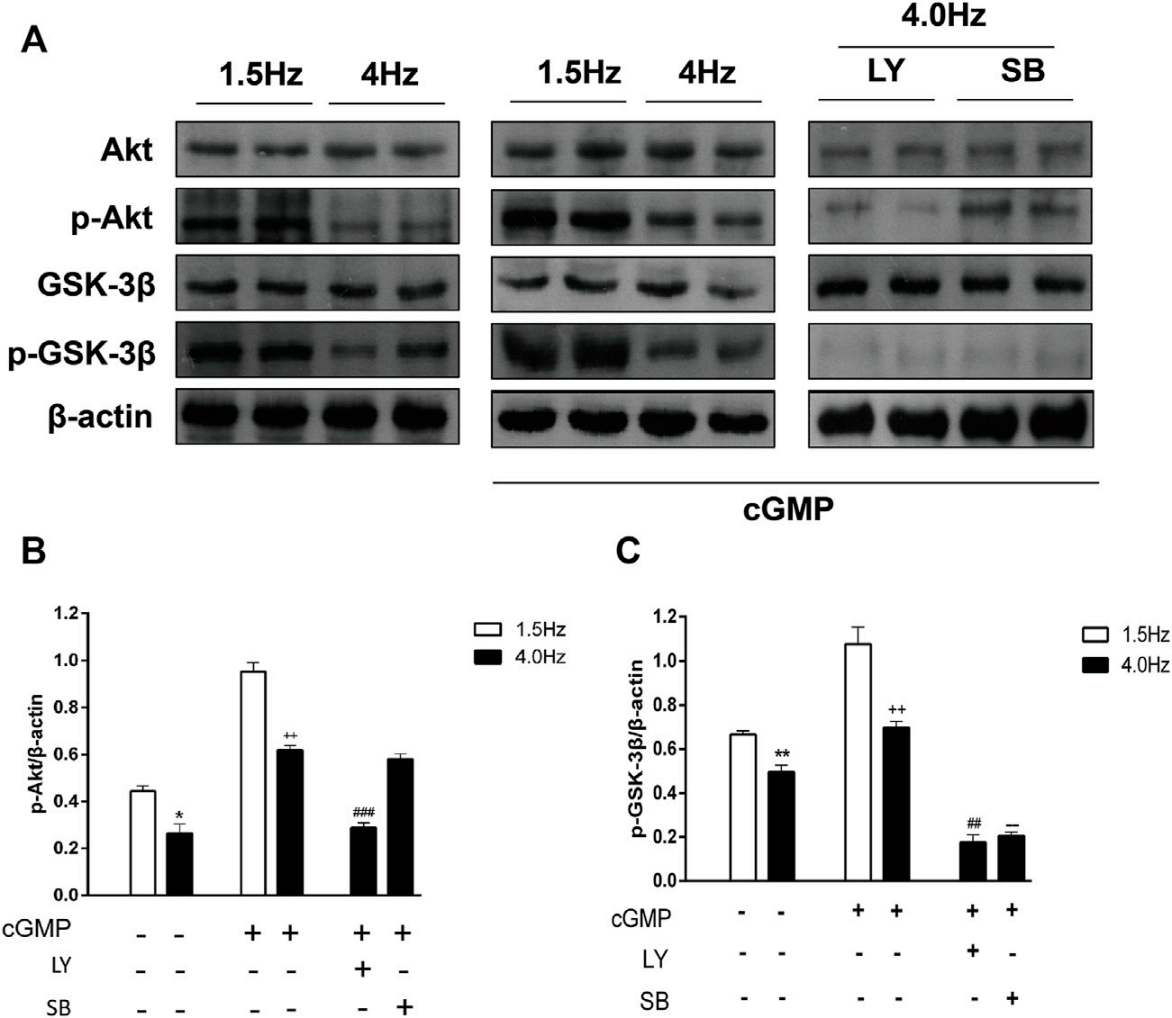
Effects of high-frequency stimulation on the cGMP/Akt/GSK-3β signaling pathway. **(A)** Effects of high-frequency stimulation, cGMP, and inhibitors on the Akt, p-Akt, GSK-3β, and p-GSK-3β protein levels were detected by Western blotting. **(B)** Quantitative analysis of p-Akt protein levels by following high-frequency stimulation, cGMP treatment, and inhibitor treatment. *p < 0.05 vs. 1.5 Hz; ^++^ *p* < 0.01 vs. 4.0 Hz; ^###^ *p* < 0.001 vs. 4.0 Hz + cGMP. **(C)** Quantitative analysis of p-GSK-3β protein levels following high-frequency stimulation, cGMP treatment, and inhibitor treatment. **p < 0.01 vs. 1.5 Hz; ^++^ *p* < 0.01 vs. 4.0 Hz; ^##^ *p* < 0.01 vs. 4.0 Hz + cGMP; ^--^ *p* < 0.01 vs. 4.0 Hz + cGMP. Data are expressed as mean ± SEMs, *n* = 6.

To determine the changes in the cGMP/Akt/GSK-3β pathway after the isolated atrial perfusion experiment, we detected the Akt/p-Akt and GSK-3β/p-GSK-3β protein levels in atrial tissues by Western blotting. We found that Akt and GSK-3β phosphorylation levels were decreased significantly in atrial tissues from rabbits in the high-frequency stimulation group compared with that in the control group (Figure 11). After adding cGMP, Akt, and GSK-3β phosphorylation levels were higher than that in the control group (Figure 11). Furthermore, the Akt and GSK-3β phosphorylation levels were decreased after adding LY294002 and cGMP to the high-frequency stimulation group. When SB216763 and cGMP were added to the high-frequency stimulation group, the phosphor-Akt did not change; however, phosphor-GSK-3β was decreased (Figure 11). These results were consistent with the results from the RAP model, suggesting that the cGMP/Akt/GSK-3β signaling pathway was inhibited, and ANP secretion was regulated by the cGMP/Akt/GSK-3β signaling pathway during RAP.

## 4 Discussion

In the *in vitro* perfusion model, we used 1.5 and 4.0 Hz stimulation, respectively, these simulate normal heart beats and heart beats of atrial fibrillation. It was found that normal physiological activity of the heart could be maintained in isolated atria when stimulated at 1.5 Hz. When we used 6, 8, and 10 Hz to stimulate atrial we found that the isolated rabbit atria showed significant rigid behavior and were no longer able to perform subsequent experiments. We found that the best experimental effect was achieved with 4 Hz frequency stimulation, and we finally chose 4 Hz.

Clinical studies have shown that the concentration of ANP is increased in patients with AF (Kurosaki et al., 2007). We observed that ANP levels increased significantly by high-frequency stimulation, consistent with the results of clinical studies. ANP is synthesized in cardiomyocytes and then released into the extracellular fluid under the effects of the mechanical stretch of cardiomyocytes, followed by spread into cardiac blood (Cho et al., 2002). The synthesis of ANP by atrial myocytes is enhanced during AF, due to ineffective atrial contraction, blood stasis, atrial volume expansion, and tension increase. At the same time, ANP in the extracellular fluid of atrial muscles enters the blood in the atrial cavity because of irregular contraction activity in the atrium. Moreover, in rapid pacing experiments of the atrium, plasma ANP levels are higher than normal, and other *in vivo* studies have shown that the ANP level is related to disruption of cardiac structure and function, representing an important biological marker of heart failure (Perez et al., 2010).

NPR-A includes the extracellular domain, kinase domain, and intracellular domain. The kinase domain is the center of regulating guanylate cyclase activity and receptor sensitivity. ANP combined with NPR-A extracellular domain to activate PGC and increase cGMP production. However, in our study, we found that ANP secretion was increased, whereas the cGMP concentration was decreased during rapid pacing. In pathological states, such as heart failure (Bryan et al., 2007) and hypertensive heart disease (Pandey, 2019), NPR-A is damaged, which reduces the activity of ANP-dependent GC (Pandey, 2014). In our study, NPR-A was less sensitive to ANP and PGC activity reduction after 8 h of rapid pacing stimulation, although the expression of NPR-A did not change. The activity of GC is then affected, resulting in decreased cGMP concentrations in the atrium and affected downstream signaling molecules. In this study, we observed that rapid pacing led to atrial mitochondrial damage, which influence ATP production and GC activity as ATP can enhance GC activity (Duda, 2010). RAP leads to mitochondrial DNA damage in atrial myocytes and the impairment of mitochondrial oxidative phosphorylation function (Pool et al., 2021), it eventually result in the disorder of ATP synthesis and the imbalance of energy metabolism in atrial myocytes, which may be one of the reasons for the decrease of PGC activity. Therefore, the sensitivity of NPR-A to ANP and PGC activity decreased during RAP, resulting in reduced cGMP production. However, further studies are needed to assess the specific mechanisms through exact cGMP levels and the cascade reaction of downstream signaling molecules in atrial tissue during AF.

Akt and GSK-3β, as downstream signaling molecules of cGMP, play important roles in the cardiovascular system. Our findings showed that the adding of signal transduction inhibitors in the isolated atrial perfusion model attenuated the inhibitory effects of cGMP on ANP secretion. One of the functions of Akt in cells is to promote cell growth; indeed, short-term activation of Akt can lead to physiological cardiac hypertrophy, although long-term overexpression results in pathological myocardial hypertrophy (Shimizu and Minamino, 2016). Moreover, GSK-3β is involved in myocardial reperfusion injury (Ghaderi et al., 2017). Thr308 and Ser473 are important phosphorylation sites in Akt (Abeyrathna and Su, 2015), upon phosphorylation by upstream signaling molecules, Akt is activated and can carry out its various functions. Unlike Akt activation, GSK-3β phosphorylation occurs at Ser9, which results in inactivation of the kinase (Sharma et al., 2020). Thus, GSK-3β is regulated by modulation of Akt phosphorylation, enabling regulation of downstream signaling molecules in the cardiovascular system.

**FIGURE 12:**
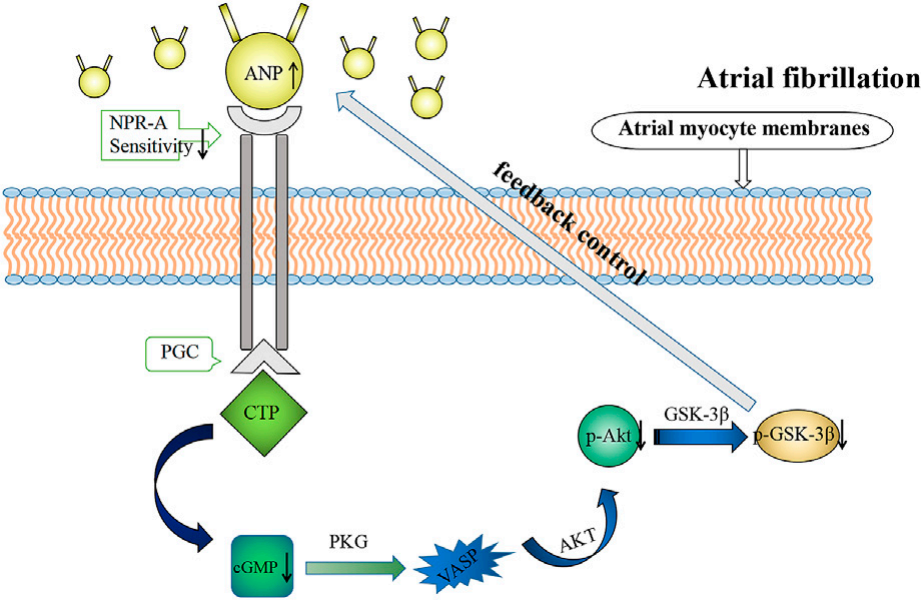
Summary figure.

In addition, cGMP is involved in the regulation of atrial dynamics (Zhao et al., 2016) and cardiac endocrine signaling (Lee et al., 2000). Here, we demonstrated that cGMP inhibited ANP secretion and atrial dynamics in rabbits in the isolated atrium high-frequency group, which is consistent with the negative inotropic effect caused by cGMP. cGMP-dependent protein kinase K (PKG) is the most important signaling molecule on the downstream of cGMP and can regulate Ca^2+^ concentration in the myocardium through the cGMP/PKG signaling pathway, exerting negative inotropic effects on the myocardium (Fellner and Arendshorst, 2010). cGMP can also exert negative inotropic effects on the myocardium through cGMP-dependent protein kinase (Wegener et al., 2002). Therefore, during RAP, NPR-A activity reduces and cGMP production decreases; the Akt/GSK-3β pathway was inhibited and the inhibition of cGMP on ANP was weakened, resulting in increasing compensatory ANP secretion (Figure 12). It is conceivable that RAP could eventually facilitate the maintenance of normal blood volume and arterial pressure.

## 5 Conclusion

We used the rabbit model to demonstrate connections between ANP and cGMP during RAP. We elucidated ANP increasing and cGMP decreasing due to the decreased sensitivity of NPR-A to ANP and PGC activity. In addition, cGMP inhibited ANP secretion and atrial dynamics in an isolated atrial perfusion model of high-frequency stimulation, and Akt and GSK-3β inhibitors decreased the inhibitory effects of cGMP on ANP secretion. We uncovered that ANP secretion was regulated by the cGMP/Akt/GSK-3β signaling pathway during AF. These findings provide important insights into the mechanisms mediating ANP secretion and cGMP concentrations in AF.

## Data Availability

The original contributions presented in the study are included in the article/Supplementary Material; further inquiries can be directed to the corresponding authors.
